# Observations on the Proximate Cause of the Intermitting Pulse, and on the Forces Producing the Venous and Absorbent Circulations

**Published:** 1861-05

**Authors:** Thomas Carroll


ART. I.—*Observations on the Proximate Cause of the Intermitting Pulse, and on the Forces producing the Venous and Absorbent Circulations.* Read before the Cincinnati Medical Society, July 5th, 1859. By THOMAS CARROLL, M.D.

IT is now more than thirty years since I began to observe with care the phenomena attending the intermitting pulse, and their probable cause; at a somewhat later period, my attention was arrested by a few facts to a careful observation of the venous circulation. Several cases were observed in which the pulse intermitted through a longer or shorter period. In one of these the pulse was subject to constant intermissions through a period of ten years. In this instance, one intermission took place every fourth pulsation, with but little variation. When death occurred, no examination was made of the body; but it was thought that tuberculosis had produced the fatal result. A far more probable cause, however, was a diseased condition of the heart, or of its valves. When a student, I observed, in a man thirty-five years of age, the intermitting pulse through a period of several weeks. At the time, the patient was laboring under rheumatic fever, from which he recovered; he was also relieved of the intermitting pulse, but died a few years after. These, with other cases, produced a strong desire to discover the causes which brought about such results.

The remote cause could easily be supposed to be nervous irritation, etc.; but the immediate or proximate cause seemed to me a mystery which could not be unraveled by anything I then knew. At first view, the knife and the dead body appeared to be the only means by which it could be reached; but what could be here told, when the heart had become motionless?

I knew that the hearts of living animals had been exposed, and the action of the auricles and ventricles observed; but the hurried circulation of a heart, thus exposed, was not calculated to instruct the observer in the true nature of the venous circulation. Hence, but little had been observed which could increase our knowledge on this subject. Under these circumstances, I concluded to make a series of observations on exposed hearts whose actions should be partially suspended, and, if possible, rendered much less frequent than when in health. To effect this, a few drops of prussic acid were dropped on the tongue of a cat; and in a few moments the animal became motionless. I now immediately cut down on the pericardium, extended the incision through the diaphragm, and exposed partially the abdominal viscera. The pericardium was then slit open, and one of the auricles freely exposed. The heart was found to still act, and to continue to do so for about half an hour. This circumstance gave me great pleasure, as the slowness of the action, I thought, would afford ample opportunity for careful and accurate observation. There were but few strokes of the ventricles during a minute, and these became less frequent until they ultimately ceased.*

I observed, however, that although this action of the ventricles became less frequent, the diminution was not perfectly regular; but that, after the ventricles had labored awhile, intermissions of them occurred every few strokes. But this was not the case with the auricles, for their action remained steady; and although their contractions became less frequent, yet there was the most perfect regularity to the last in this diminution, until, finally, they made a last effort to stimulate the ventricles to further contraction.

Now, it is evident that the ventricles, in all cases of intermitting pulse, lose at each intermission the time of one ventricular contraction, or nearly so. Not so, however, with the auricles; for they labor on or contract as usual. This regularity is less or more frequent, owing to rest or motion, or other circumstances; but still they never intermit as the ventricles do.

It is, I believe, conceded that the auricles, by their contraction, fill the ventricles with blood, and the ventricles are by this means forced to contract and throw their contents into the aorta and pulmonary artery; and that these contents are prevented from returning during their quiescence by the semilunar valves. At least this view of the action of the auricles is believed by nearly all physiologists whose opinions are worth anything. After this round of contraction and relaxation of the ventricles and auricles, another succeeds, and continues indefinitely; and in health these contractions are regular—that is, the contraction of the ventricle regularly follows that of the auricle. But this harmony of action is occasionally disturbed by some abnormal cause. The auricles contract and fill the ventricles; but the latter disobey the stimulus of the blood forced into them, and do not contract. Now, what takes place? The auricles have thrown most of the blood which the cavas and pulmonary veins had sent them into the ventricles. The cavas and pulmonary veins have no more power of filling the auricles, and of

---

* When making these experiments, I laid bare the intestines in several cases, and found that by pricking them with a sharp instrument the peristaltic action was beautifully excited, running from above downward, and rendering the bowel as hard as a whipcord.

course life must cease unless they are again stimulated, from some source, to contraction. Death, however, is prevented by the fluid that has just been thrown into the ventricles flowing suddenly back into the auricles: for when the ventricles do not contract, the bicuspid and tricuspid valves remain perfectly quiet, resting on the walls of the ventricles; thus leaving the blood which had been forced into the ventricles free to flow back, which it immediately does, and fills the auricles, which are thus again stimulated to contract. And now the ventricles no longer resist, but contract, and continue to do so for a longer or shorter time, according to circumstances; generally the regular contractions occur two, three, or more times, and then another intermission takes place. This recurrence of intermission sometimes continues during a considerable time: and it may now be said that the action of the heart is in an abnormal condition, or, in other words, that the subject of it has intermitting pulse, or an intermitting ventricular action. It is here a matter of importance to know that the cavas and pulmonary veins have no power of refilling the auricles, as I have observed; that the ventricles after receiving the blood from the auricles were not thereby sufficiently stimulated to contract; yet as their valves remained quiescent, the blood flowed back into the auricles, which were in a relaxed condition, and by their contraction had produced a condition in their locality bordering on a vacuum, so that the blood, in finding an equilibrium, restimulated the auricles. But, as has been stated, no part of this stimulation grew out of blood sent from the cavas and pulmonary veins. When the auricles were thus stimulated by the return of blood from the ventricles, they contracted again, and now succeeded in forcing the ventricles to act; and the blood, instead of being thrown back to the auricles, took its wonted course through the arteries. Now, the auricles became again filled; but this was done by fluid from the cavas and pulmonary veins.

We have now reached a point which, if we can clearly understand how the cavas and pulmonary veins are enabled to fill the auricles, must be of the greatest moment to the interest of physiology; because here lies the great secret of the power by which the venous circulation is carried on. As has been said, the blood that stimulated the auricles, after the intermission of the ventricular contraction, came from the cavas and pulmonary veins, but no drop appeared to swell the auricles until after the contraction of the ventricles; then the auricles were suddenly filled by a wave of blood from the cavas and pulmonary veins. This wave was obviously produced by the stroke and swell of the arteries.

My observations during my examinations were confined mostly to the ascending cava and the descending aorta, as these were brought more into view; hence, I saw that the moment the wave of blood was propelled along the artery, the auricle was filled by a sudden jet of blood forced from the ascending cava. This propulsion caused an instant contraction of the auricle. Hence we must now consider the facts which, connected with the venous circulation, demonstrate the cause of the motion of the blood in the veins.

It has been seen, in the course of this essay, that the wave of blood passing through the great arteries causes the auricles to be filled. How this wave of blood has the power of sending that of the cavas and pulmonary veins into the auricles and distending them, is the great question to be solved with regard to the venous circulation. We shall, for this reason, take a view of the location of the large arteries and veins, and of the tissues immediately connected with them. These tissues are bound up, in a common sheath, with the greater arteries and veins; and these again are surrounded by other tissues, more or less dense; and all are supported, as matter in general, by the weight of the atmosphere.

Now, let us admit that the descending aorta and the ascending cava are in their usual position; that the vena cava is filled with blood, or nearly so, and that the aorta has discharged most of its contents; that now the heart, to bring about an equilibrium, contracts, and throws an ounce of blood into the artery which is in juxtaposition with the cava, and both surrounded by dense tissues, it is then obvious that the fluid in the vein must be forced out of its place by the wave that is propelled along the artery. The blood in the cava is prevented from a retrograde motion by the valves in its ramifications, and by other causes, such as the constant supply that is passing through the valves and along the veins; hence, the blood acted on by the swell of the aorta rushes by the distended artery, and finds room in the quiescent auricle and ventricle. The auricle now, however, by distention stimulated to contraction, sends more blood into the ventricle, and urges, by the quickness and force of its stroke, the action of the ventricle. Now another ounce of blood is again forced along the artery by the ventricle of the left side, the artery again lashes the cava, and with the aid of the descending cava again fills the auricle, and it again relieves itself as before.

As this wave of blood progresses in the artery, it sends, of course, nearly a corresponding amount in the opposite direction; room for which is made by the lessening of the calibre of the artery, by its contraction in forcing onward the wave of blood, so that the blood in the vein finds an exit on the side next the heart.

The venous blood, at a distance from the heart, and in the smaller vessels, has rather a continuous than an intermitting motion. This is especially the case where veins are unaccompanied by arteries; but even in situations where veins are not accompanied by arteries in immediate juxtaposition, the blood flows in them occasionally by jets. This I have observed more than once at the bend of the arm, in plethoric subjects, when I had opened a vein in that locality. The proximity of the arteries and veins in this part is such that, where there is much tension, the flow of blood from the veins will occasionally demonstrate the arterial stroke by flowing in jets.

Now, we know that in the veins of the extremities there are numerous valves, especially after they dip beneath the fascia, and that these valves prevent a retrograde motion of the blood in those veins. The arterial blood, carried along the arteries beyond this bulwark of the veins, forces their contents into the venous capillaries, and the venous blood receives an impulsive motion by the swell and stroke of the numerous small arteries, and is by this means hastened through the valves, which now become a barrier against its return; and now the larger veins approximate more closely, and are finally bound up in the same sheath with the great arteries of the economy. These large arteries, of course, constantly swell and relax—lashing the veins bound up with them, the fluid in which, finding no way of returning behind the valves which it has passed, seeks another outlet, which it finds in the empty and quiescent auricles. This entrance of blood into the auricles is, as we have seen, produced by the stroke of the descending aorta on the ascending cava—each swell and stroke of the artery filling, in conjunction with the supply from the descending cava, the right auricle. If, then, this condition of the circulation exists in the neighborhood of the heart, it must be obvious that it exists throughout the entire circulatory system.

The reader will here recollect that I have confined my reasoning mostly to the descending aorta and ascending cava. It is, however, intended to apply to the ascending aorta, the pulmonary arteries, and descending cava. The forces which carry on the circulation through the pulmonary veins are, undoubtedly, of the same kind as those that we have described as carrying on the general circulation. The pulmonary artery and its ramifications are placed in the most advantageous situation for forcing the arterialized blood through the pulmonary veins toward the left auricle. The left pulmonary veins cross in front of the descending aorta, and behind the pulmonary artery, and reach the left auricle, and at the moment the ventricles are contracting throw their blood into the auricle; while the right pulmonary veins are aided in discharging their contents into the auricle by their proximity to the aorta immediately below the arch. The shortness of the pulmonary arteries and veins aid in the rapid circulation through the lungs. Collapse of these organs is much prevented by the bronchial tubes, whose rings, and general cartilaginous structure, with the air constantly in them during expiration, and the weight of the atmosphere during inspiration, give much support to these vessels. These forces probably equal the defenses belonging to the venous system in general, by the tonicity of their coats, and by the dense tissues immediately surrounding them, as well as by those still more distant, so that the powers of the heart and arteries are about equally supported in every part of the body exterior to the bones and tendons.

We know that when the valves of the veins, by disease, over-exertion, or by a long depending position of a limb, lose their power of closing and sustaining their column of blood, a serious disease is the consequence.

But if we review what has been said in the foregoing pages, with regard to the forces which carry on the venous circulation, I think it will appear that, so far as induction can prove anything, it has been done: firstly, in the action of the heart causing the swell of the arteries; and secondly, that that swell causes the veins to fill the auricles. However, to put the matter at rest, I shall bring to my aid the following experiments:—

I gave, with the assistance of my friend Mr. Scott, to a medium-sized dog enough of prussic acid to suspend animation; I then cut down rapidly on the heart, and opened the pericardium, and exposed most of the right auricle and part of the left. I then made an incision about a quarter of an inch long and a little to the right of the auricular appendix, while the heart was yet acting strongly. The moment the incision was made, the blood was forced out six or eight inches in jets in obedience to the swell of the arteries, and not by the contraction of the auricle, for that was passive. A jet of blood from the orifice in the auricle occurred at each contraction of the left ventricle, for the right did not act. This experiment demonstrates the accuracy of all the conclusions I had arrived at before, because the force of the blood sent along the cavas had obviously power enough to fill the auricle; but it now seemed clear that the blood not only stimulated the auricle, but in a great measure filled the ventricle, and all that the auricle seemed to do was to stop the jet of blood which was forced into it, and by a sudden contraction to give an impulse to the ventricle which again made it contract; for I observed that the auricle by contraction did not materially lessen the amount of blood in it, but simply produced an impulse in the amount already in it and the ventricle, so that the organ was stimulated to contract.

As a confirmation of the foregoing circumstances, I shall report the following case: I gave a cat of a large size enough of prussic acid to cause sudden quiescence. My friend Mr. Scott cut down on the pericardium and slit it, so that the auricles were exposed; but, owing to the intense impression of the poison, and a little longer delay than usual in opening the pericardium, the heart had ceased to beat. The auricles were much congested, and indeed the ventricles seemed to be full of blood. The hope of any action seemed at an end. I, however, made a small incision into the right auricle, just at the root of its appendix; blood now flowed in a continuous stream for a few moments—then, to our great astonishment, the contraction of the left auricle and left ventricle began, and blood was thrown in jets from the incision at each contraction of the ventricle during eight or ten pulsations.

I have mostly, in describing the action of the auricles, followed the generally received opinions, that their contraction fills the ventricles; but this, as I have shown, is only partially true. It would, indeed, seem that the auricular contraction is only useful to stop the jet of fluid sent by the veins into them, and to direct the current of blood with force enough to excite the ventricles to contract.

If we consider the formation of the auricles, we must come to the conclusion that they are badly calculated to throw any considerable amount of blood into the ventricles. They have no valves to close at the termination of the veins, so that if they were to empty themselves completely, they must do it at a great disadvantage, as there would be no barrier between them and the termination of the veins. We find the ventricles, defended on the one hand by the bicuspid and tricuspid valves, and on the other by the semilunar valves, have the power of very nearly emptying themselves; but the auricles are in a great measure cul-de-sacs, with large and undefined openings, which have at every systole to receive a renewal of fluid with a force that answers all the purposes of valves, and even more, as it not only fills them, but in a great measure the ventricles too. Without this rapid current, valves would be of the last importance. So, then, it would seem that the principal use of the auricles, as has been said, is mostly to direct the current of blood into the ventricles, and then a contraction, more or less severe, stimulates the ventricles to action. The following experiments will elucidate how much the swell of the arteries has to do with the venous circulation.

I gave a large dose of prussic acid to a cat, so that the natural action of the heart had ceased before I could observe its pulsations. I, however, found that the auricles were quite distended but did not contract. I then, in order to prove that the current of blood sent along the veins was caused by the swell of the arteries and no other force, made a small incision close to the root of the auricular appendix. The blood now flowed out in a quiet stream, rising about two inches from the incision; this was evidently produced by the tension of the veins and auricles; there was now no jet.

These experiments seem to prove, beyond the possibility of a doubt, the accuracy of my reasoning with regard to the forces causing the venous circulation.

I recently suspended the animation of a cat, cut down and opened the pericardium so that the right auricle and part of the left were exposed. In making this dissection I opened both pleuræ, the effect of which was to produce an immediate collapse of both lungs, and to cause a sudden withdrawal of the supply of blood from the left auricle, and, of course, an early cessation of the action of that organ; but as it acted for a short time, though on a very small amount of blood, it still produced a few contractions of the left ventricle. The right auricle still continued to act and to stimulate the right ventricle, but owing to the complete quiescence of the left ventricle, it could not empty itself of its contents, which was probably owing to the fact that the valves of that organ had not the power of closing the communication between it and the auricle. It was, however, convulsed for a short time, owing probably, or even certainly to the considerable amount of blood it contained. This convulsive action continued for a minute or two after the cessation of the action of the auricle, but at no time amounted to a full contraction.

This experiment shows that one ventricle may act, though in an imperfect manner, while the other has become entirely quiescent; but, of course, this must be of short duration. It, however, proves that both the left and right ventricles may act independently of each other. Another circumstance occurred in the contractions of this heart worthy of notice, which was, that during a few contractions of the ventricles, they acted twice, independently of the action of the auricles; then again the auricles had to contract several times to produce another ventricular contraction. This variation in action from what I had often seen, seemed to be produced by the sudden cessation of the circulation through the lungs, due to their collapse, from the admission of atmospheric air within the pleuræ. Still the same intermissions occurred in the ventricular action, and were only relieved by unceasing contractions of the auricles.

These experiments seem to close the avenues of doubt with regard to my reasoning on the forces which carry on the venous circulation, and the abnormal causes of the intermitting pulse. I could only now wish for an experiment on the right auricle by making smaller incisions to see how far the blood would be thrown by each swell of the arteries; but this is at present out of my power; and I must, therefore, leave it to others, or to a future time.

We now come to consider the portal circulation; and, in order to have the matter properly before us, we must regard, for a moment, the anatomical, or, rather, physiological construction of the portal system, as well as some of the parts immediately connected with it.

The portal vein may be compared to a tree, with its roots fixed in the liver, and its branches spread out on the mesentery and intestines. The trunk is short, and, like all the branches, has no valves, so that the blood moves in it with ease. The ramifications in the liver are preserved against external pressure by the dense and unyielding substance of that organ, so that no impulse from without, made by arterial pulsation, can affect its circulation; but the hepatic artery, in its ramifications, traverses the same channels, being bound up by the capsule of Glisson in company with this vessel and the hepatic duct, so that the flow of blood in the artery has a direct influence on the duct and vein bound up with it. And this influence extends throughout their branches; for, there being no valves in the portal vein, the blood hurries on, without resistance, to the radicals of the vessel. Now, the hepatic artery sends, at each pulsation, a certain quantity of fluid into the liver, which must have room made for it. The blood in the branches of the portal vein must, of course, yield in some direction; and, there being no valves to obstruct its motion toward the radicals, it takes that direction, because the calibre of the ramifications, taken together, is much greater than that of the trunk. But still another obstruction to its return, and one that is irresistible, is the stroke of the abdominal arteries, without the liver, which subjects all the branches of the portal vein to their action, unless, indeed, it be those within the spleen and pancreas, which are defended, in some degree, by their organism, from this influence. The abdominal muscles, with the diaphragm, and peristaltic action also, aid in pressing the blood toward the liver; and hence there is a material difference in the mode by which the portal ramifications without the liver, and those within it, are acted on by external causes.

But, to make the matter more clear, let us recapitulate, partially, what we have shown. The ramifications of the portal vein are acted on by various causes. The motion of the body acts on them; the muscles of the diaphragm and abdomen produce alternate tension and relaxation; and, independent of all this, comes a power which especially causes this blood-vessel to act the part of an artery. Seventy times every minute an ounce of blood passes through the descending aorta, and much of it, of course, through the abdominal arteries. The swell of these arteries must have space made for the fluid thus increased in quantity. This space is made by the flow of blood through the ascending cava, which has to discharge a certain amount through the diaphragm at every pulsation; and the portal vein, too, being now lashed by each pulsation of the abdominal arteries, throws a portion of its contents through its wide-spread ramifications in the liver, whose united diameter constantly increases from its first bifurcation. The hepatic veins have walls so constructed that the arteries within the liver and the ramifications of the portal vein have but little power in forcing their contents onward; but as they have comparatively unyielding walls, the action of the descending aorta, after they emerge from the liver, urges them to throw their contents into the vena cava ascendens. These veins no doubt have a constant pressure on them caused by the blood pressed into the liver by arteries and portal veins.

Calculations show that about 260 pounds of blood pass the abdominal aorta and its branches every hour; and that no less than 6240 pounds are sent through them every twenty-four hours. These realities show that an immense amount of blood passes through the blood-vessels of the abdomen, on its way from the heart; and prove that a similar amount must be sent toward the heart by the ascending cava, much of whose blood is received from the hepatic veins.

The ultimate radicals of the portal vein have, no doubt, much to do with the easy transmission of the blood through its ramifications in the liver. We here find a curious circumstance in the formation of the gall-duct and gall-bladder; for it would seem that the bile, after its formation, has no power in general of finding its way back into the circulation, while the influx of arterial blood constantly urges its flow along the biliary ducts, until it emerges from the substance of the liver, and is then either forced through the hepatic duct until it is discharged by the ductus choledochus into the duodenum, or finds a resting-place in the gall-bladder.

I propose now to show that the fluids in the absorbent system are carried through them by the same forces which carry on the venous circulation; and that this is particularly the case in the thoracic duct. Now this duct lies between the descending aorta and vena azygos, and runs close to the left carotid, until it descends to enter the venous system. It is supplied with numerous valves, and is so fixed that it is constantly lashed by the arteries among which it lies. This is, however, especially the case with regard to the aorta and left carotid; for the swell of these vessels press it at every pulsation, so that its contents are forced in all directions, but can only find an avenue for escape in its motion toward the left subclavian vein; for when a drop of its contents once passes a valve it can never return. Of course a constant stream is directed toward its final termination, where, by proper experiment, it may be seen dropping its contents, drop by drop, evidently being urged forward by each pulsation of the aorta. Can, indeed, there be any other method by which the absorbent system can perform its functions, than by the impulse which is given by the swell of the arteries?

My last argument with regard to the direct or motive power by which the venous circulation is carried on, is that of the circulation of the brain.

This organ is situated in a bony case, which in the adult is unyielding. The only inlets and outlets are those of the arteries and veins, and these, we know, ramify throughout the organ and are constantly full; that is, there is in the brain no empty space. The arterial blood may, indeed, be greater at one time than another, and so may the venous be less or greater as the arterial is increased or diminished; or there may be an atrophied condition of the brain, which may require greater distention of the arteries. Still, the concavity of the cranium must throughout life be full.

This being conceded, proves that a single drachm, or even a drop of blood being admitted by the arteries, must of necessity displace an equal amount of venous blood. The question then is, how is this effected? We know that the longitudinal and lateral sinuses are constantly filled with blood even after the extinction of life; and we know also that they are supplied by numerous veins, some of which enter so that their contents cannot return to the brain. Now, when the stroke of the arteries occurs, and forces into the capillaries of these veins a certain amount of fluid, their sides are lashed by the arterial impulse acting on the brain, and a corresponding amount of blood is forced into the sinuses. The extremities of the sinuses rest in a fossa on either side, and have a free opening into the internal jugular veins. These lie throughout their entire length in a common sheath with the carotid arteries. Now, at the moment that the carotids swell with blood, they drive in an opposite direction the blood in the jugulars; so that just before the brain receives an impulse from the arteries the jugulars have been relieved of an equal amount of their contents, and are again ready to receive and actually invite a flow of blood from the sinuses. Thus in succession does the brain receive and is unburdened of its circulating fluid, nearly on the same principle or method as that controlling the motion of blood in the arteries and veins in the immediate neighborhood of the heart.

It may not be improper to refer here to physiologists who have written on the subjects contained in this essay, though I think extended quotations unnecessary. Among those most worthy of notice, the immortal John Hunter stands foremost, because he approaches nearer the truth than any other writer I have examined. Hunter observes that "there is a pulsation in the veins; for," says he, "when we bleed a patient on the hand or foot, we see a strong jet—much more in some than in others, and much more here than in the bend of the arm. The query is, does this arise from the immediate stroke of the heart, or is it by the lateral pressure occasioned by the swell of the arteries? To ascertain this the better, it is necessary to observe several things. We may remark that the pulsation in the veins is more in some parts than in others; thus, I should suppose it was more in the veins of the kidney, spleen, lungs, and brain, especially the last, than in many other parts; but this, from the lateral swell of the arteries, cannot from the above observations affect all parts alike; for the veins on the back of the hand being superficial, and not surrounded by vascular parts, could not be affected by arteries, and this acceleration given to the blood's motion in the smaller veins is carried to those on the back of the hand. But I think I have seen the difference in the projections so great, that it hardly could arise from that cause alone; and, indeed, if this was the only cause, we should have it in some degree in every vein; for every vein is so far surrounded as to be in some measure affected from the swell of the arteries of the part; but we certainly do not perceive it in so great a degree in the bend of the arm. The large veins near the heart have a pulsation which arises from contraction of the heart, preventing the entrance of the blood at the time, and producing a stagnation. This I saw very evidently in a dog whose chest I opened, and produced artificial breathing; but I could not say whether this arose from the contraction of the auricles, the ventricles, or both."

Now, in this quotation, we find this remarkable statement: that "the larger veins near the heart have a pulsation which arises from the contraction of the heart, preventing the entrance of the blood at the time, and producing a stagnation." Were this true, that is, that the blood in the veins experiences a stagnation when the heart contracts, there would be no possibility of the auricles being filled; because, it is only when the ventricles contract that the auricles can be replenished with blood; for, at the termination of the contractions of the ventricles, the auricles must be ready to refill the ventricles. This preparation of the auricles has taken place from the contraction of the heart, which becomes lessened for the moment; this lessening of the heart by contraction consequently causes a swell of the arteries, which swell forces the blood in the cavas and pulmonary veins to flow into the auricles, which now become stimulated to contraction. Of this, however, I have before spoken. From these remarks of Hunter, it would seem plain that he had not a perfectly clear idea of the venous circulation, although he observed much that was true, and only wanted further investigation and the discovery of facts to come to accurate conceptions of the matter.

But what must we say of the physiologists who have followed Hunter? Certainly not that they have added clearer notions of the venous circulation to our knowledge; but, rather, that they have not progressed a single step, if, indeed, they have not really rendered the subject more unfathomable than it was.

Dr. Carpenter does not, so far as I recollect, notice the opinion of Hunter on this subject; nor does any other physiologist, whose works

I have examined. Carpenter states, with regard to the venous circulation, that "the movement of the blood through the veins is, without doubt, chiefly affected by the vis a tergo or propulsive force which results from the action of the heart and arteries, and from the additional power generated in the capillary vessels. This is shown by the immediate arrestment of it which takes place when these forces are suspended." Now, this is a conclusive way of accounting for the cause of the flow of venous blood, but leaves us about where it found us; and, certainly, with very few more just conceptions of the particular manner, or power, by which this flow is effected. We might say, that unless the heart acted, venous blood would not continue to reach that organ; and we might say it with great propriety; but we would be left still ignorant of the real force or cause.

Anterior to, and at the time of Harvey, the liver was believed to send out the blood to the different parts of the body; but the true nature of the circulation was left to be demonstrated by that great man. The movement of the blood in the portal system is still, in the eyes of medical men, a mystery; yet all believe, and know, that blood is circulated through it.

There are opinions of physiologists which favor the notion that suction aids materially in carrying on the venous circulation. This notion is founded on a supposition that the auricles possess the power of dilatation, so that either a vacuum, or an attempt at it, draws the blood from the veins into the auricles. It has, however, been shown by Dr. Arnott, that this suction power is impossible. We think we have shown that the only power possessed by the auricles consists in repose and contraction, or, rather in contraction, as repose may not be considered a power. This action and repose of the auricles, we have most assuredly observed; the former being caused by the rush of blood from the cavas and pulmonary veins, the latter being caused by the condition of the heart. Another cause of contraction of the auricles is abnormal and only occasional; that is, when the contraction of the auricles fails to stimulate the heart to contraction, there being no renewed flow of blood from the cavas and pulmonary veins. The tonicity of the heart throws the blood back in the auricles with sufficient force to restimulate them; but of this we have said enough.

Other theories with regard to the forces which cause the venous circulation, or act as powerful auxiliaries, are founded on the action of the diaphragm, and other muscles which cause inspiration. The muscular system in general is also added to aid in this matter. There can be but little doubt that all these forces act to a small extent in aiding the venous circulation; but they act with equal, if not greater force, in aiding the arterial circulation.

We cannot close this essay better than by quoting the opinions of Laennec and Stokes, on the action of the heart and the peculiarities of the intermitting pulse. The former says that "at the moment of the arterial pulse, the ear is slightly elevated by an isochronous motion of the heart, which is accompanied by a somewhat dull though distinct sound. This is the contraction of the ventricles. Immediately after, and without any interval, a noise resembling that of a valve, or a whip, or the lapping of a dog, announces the contraction of the auricles. (I make use of these trivial expressions because they appear to express better than any description the nature of the sound in question.) This noise is accompanied by no motion perceptible by the ear; and is separated by an interval of repose from the duller sound and motion indicative of contractions of the ventricles, as it were to interrupt abruptly. The duration of the sound, and, consequently, the period of contraction of the auricles, is less than that of the ventricles,—an incontestable fact, of which Haller entertained doubts. Immediately after this systole of the auricles, there is a very short yet well-marked interval of repose; subsequently we feel the ventricles swell anew with the dull sound and gradual progression which characterize their action; then follows the quick and sonorous contraction of the auricles, and again the renewed but momentary immobility of the heart." On the intermitting pulse, he states that "the duration of the intermission is very variable, and may seem to make this affection into well-marked varieties. Sometimes the intermission is shorter than the arterial pulsations, sometimes it is equal, and sometimes it is longer."

"The first kind of intermission is most common. It is most frequent in old age, even during health. At other periods of life, it is only observed in certain diseased states of the heart, particularly hypertrophy. By means of the stethoscope, we ascertain that this species of intermission always succeeds the contraction of the auricles."

These quotations show that Laennec observed the action of the ventricles and auricles, both in health and disease, with wonderful accuracy,— an accuracy which we think aids in proving the positions we have taken. Yet we must acknowledge that he only observed those facts resulting from sound and motion. The opinion of Laennec, that the intermitting pulse in old age may exist during health, is probably correct; and it would seem almost certain that, in the young and middle aged, the existence of this morbid action is, as he states, the accompaniment, in all or most cases, of hypertrophy. The two cases which we have noticed in the first part of this essay go, we think, to prove this, as they both fell victims to an early grave.

Professor Stokes states, in his learned work on the diseases of the heart and arteries, that "among the physical signs of the derangement of the action of the heart, he knows of none more obscure in its nature than the doubling of one of the sounds. It is as if the sound was divided into two sounds; in some cases similar in tone and duration, in others, differing in both these qualities. This sign seems to affect the left more than the right side of the heart; and, in a majority of cases, occurs in connection with the second rather than the first sound." This last part of the quotation, that is, that "in a majority of cases, it occurs in connection with the second sound," goes to prove my position,—that the auricles, in the intermitting pulse, continue their contractions, though the ventricles do not. Had this learned physician understood the true nature of the intermitting pulse when he wrote, he would not have said that "among the physical signs of the action of the heart," he knew "of none more obscure in its nature than the doubling of one of the sounds."

In the foregoing discussion, I have avoided noticing the probable motive power by which the capillary circulation is carried on, for the reason that I have come to no certain conclusion about the matter. The capillaries may have a circulatory power independent of the heart's action; but I suppose that the arteries and veins, acted on by the heart, must be the great powers which move the blood in these ultimate terminations. The nervous system, or energy, no doubt has a great influence, through the coats of the blood-vessels, in determining their calibre; and, of course, has the power of sending blood to local parts, though this power can only aid the action of the heart. The capillaries may have a still greater energy, derived from the nervous sources, which may aid them to a circulatory power. We presume much that is now believed with regard to this supposed power is more imaginative than real. The report of the following case will clearly show that the great arteries and veins are placed in juxtaposition for other purposes besides convenience, and that such proximity is essential to the venous circulation:—

A. B., aged about seventy, a plasterer by trade, who had long been in the habit of excessive fits of drunkenness, became fatally diseased, as follows: When I was called to the case, I found the patient in bed, with his legs of a venous or purple color, his arms of a less intense venous hue, and his countenance in a slightly livid condition. The pulse, at the wrists, could not be felt; but the coats of the radial arteries were hard and unyielding. This circumstance led to the examination of the arteries at different points, and I found them equally hard, and without pulsation; so that ossification of their coats was obvious. I examined the carotids, and the temporal ramifications, and the arteries of the thigh and leg and arm, but could find no pulsation—nothing but stony hardness. From this condition of the arterial tubes arose, I concluded, the whole difficulty as to the return of the blood to the heart; for that organ seemed to act with moderate force, although there were some intermissions; and no doubt many, if not all, the larger arteries within the great cavities were in a comparatively healthy condition, which allowed their action on the great veins near or more distant from the heart. I asked the patient if he could rise and walk. He said he could; and arose and walked about ten feet with much difficulty. The moment he became erect, great difficulty of breathing occurred. After he sat a few minutes, I requested him to return to bed, which he did; but his weakness seemed much increased by the effort. I found that he had been in the habit of rising occasionally; but for a long time with the same symptoms recurring. About a month after I first saw this patient, he died. His disease had lasted, in a serious condition, for some months before I saw him, and gradually increased, until it terminated in death.

Now, it would seem that this case goes far to prove that the elasticity of the arterial tubes is essential to the venous circulation; and we think it shows that the capillary system has but small share in supplying the forces which carry on the general circulation, other than what grows out of the ultimate termination of the arteries.

Since writing the foregoing pages, I have observed in the November number of the *London Lancet*, of 1859, a communication by James Nichols, F.R.C.S., "on the lateral pressure and venous circulation." This writer, again in the February number, 1860, of the *Lancet*, has another communication on the same subject. The arguments in favor of the lateral pressure and swell of the arteries, causing the venous circulation, are able; but founded, not on experiment, but on supposed results which must occur from the stroke and swell of the arteries. These views of Mr. Nichols are similar to those of John Hunter, on the same subject. Hunter, however, abandoned his views, because of difficulties which he supposed lay in the way of his reasoning.

It is clear that Mr. Nichols is a profound thinker. I, however, think I have demonstrated what he has only supposed; and extended my observations to the action of the heart itself, with its irregularities. My essay was read before the Cincinnati Medical Society, in July, 1859; and Mr. Nichols's first communication was published in the November number of the *Lancet*, of the same year.

It is also true that my discoveries were mostly made before I had seen any of Hunter's works. Both my reasoning and experiments are my own; and I regard them as putting the matter at rest as to the forces carrying on the venous, and I hope also, the portal and absorbent circulations.

